# A Systematic Literature Review of Indoor Air Disinfection Techniques for Airborne Bacterial Respiratory Pathogens

**DOI:** 10.3390/ijerph19031197

**Published:** 2022-01-21

**Authors:** Thi Tham Nguyen, Graham R. Johnson, Scott C. Bell, Luke D. Knibbs

**Affiliations:** 1 School of Public Health, The University of Queensland, Herston, QLD 4006, Australia; luke.knibbs@sydney.edu.au; 2 School of Earth and Atmospheric Sciences, Queensland University of Technology, Brisbane, QLD 4000, Australia; g.johnson@qut.edu.au; 3 Children’s Health Research Centre, Faculty of Medicine, The University of Queensland, Brisbane, QLD 4101, Australia; scott.bell@tri.edu.au; 4 Adult Cystic Fibrosis Centre, The Prince Charles Hospital, Chermside, QLD 4032, Australia; 5 Translational Research Institute, Brisbane, QLD 4102, Australia; 6 Faculty of Medicine and Health, School of Public Health, The University of Sydney, Sydney, NSW 2006, Australia

**Keywords:** bioaerosols, airborne transmission, air disinfection techniques, laboratory-based studies

## Abstract

Interrupting the transmission of airborne (<≈5 µm) respiratory pathogens indoors is not a new challenge, but it has attracted unprecedented interest due to the COVID-19 pandemic during 2020–2021. However, bacterial respiratory pathogens with known or potential airborne transmission account for an appreciable proportion of the communicable disease burden globally. We aimed to systematically review quantitative, laboratory-based studies of air disinfection techniques for airborne respiratory bacteria. Three databases (PubMed, Web of Science, Scopus) were searched, following PRISMA guidelines. A total of 9596 articles were identified, of which 517 were assessed in detail and of which 26 met the inclusion and quality assessment criteria. Seven air disinfection techniques, including UV-C light, filtration, and face masks, among others, were applied to 13 different bacterial pathogens. More than 80% of studies suggested that air disinfection techniques were more effective at inactivating or killing bacteria than the comparator or baseline condition. However, it was not possible to compare these techniques because of methodological heterogeneity and the relatively small number of the studies. Laboratory studies are useful for demonstrating proof-of-concept and performance under controlled conditions. However, the generalisability of their findings to person-to-person transmission in real-world settings is unclear for most of the pathogens and techniques we assessed.

## 1. Introduction

There are three main routes of person-to-person transmission of communicable respiratory pathogens: contact, droplets, and airborne transmission. Contact transmission can involve direct physical contract or indirect contact through a contaminated person or object (e.g., fomites). Droplet transmission involves pathogens in respiratory particles larger than ≈20 μm in aerodynamic diameter released by coughing, sneezing, talking, breathing [1], or from aerosol-generating procedures (e.g., intubation) [2]. While droplet size is a continuum and not a binary threshold, in general, droplets in this size range travel <1 m in air, settle rapidly due to gravity, and require proximity between infectious and susceptible individuals indoors. In contrast, ‘airborne’ (i.e., aerosol) transmission involves microbes in the residual droplet nuclei, after the initial droplets rapidly evaporate, which have a diameter of less than ≈5–10 µm; these nuclei can remain airborne for extended time periods and distance [3,4]. Droplet nuclei can penetrate and deposit in the tracheobronchial and alveolar airways to a greater extent than larger particles. The distinction between the droplet and droplet nuclei size ranges is indicative, as it varies under different environmental conditions (e.g., air velocity, relative humidity, temperature) [5].

Airborne transmission has attracted scientific attention for almost 100 years [6], but it has been especially topical in light of the ongoing SARS-CoV-2 (COVID-19) pandemic in the last two years. In July 2020, guidelines around the transmission of SARS-CoV-2 shifted away from the early view that it was driven by contact and droplets to reflect growing evidence that the virus can also be transmitted by the airborne route. The pandemic has re-emphasised the importance of better understanding airborne transmission in COVID-19 but also for other pathogens. For example, other viruses of public health importance, including measles [7,8], can be spread by airborne transmission indoors [9,10,11,12]. *Mycobacterium tuberculosis* (*M. tuberculosis*) remains the leading infectious cause of death globally (1.4 million deaths in 2019), and airborne transmission has long been known as the dominant transmission mode of *M. tuberculosis* [13,14,15,16,17,18]. Other respiratory pathogenic bacteria with potential, to varying extents, for airborne transmission include *Bordetella pertussis* [19], *Staphylococcus aureus* (*S. aureus*) and methicillin-resistant *Staphylococcus aureus* (MRSA), *Mycoplasma pneumoniae*, *Pseudomonas* spp., and *Streptococcus pneumonia* [20,21].

‘Air disinfection’ methods for airborne transmission seek to reduce the concentration and/or viability of microorganisms in indoor air [6]. For example, commonly used methods in healthcare settings include surgical masks (to reduce droplet nuclei formation, and also used for inward protection), respirators (to reduce inhalation of nuclei), natural and mechanical room ventilation (to dilute and remove contaminated air), filtration (to physically capture airborne organisms with filtration media), and ultraviolet-C (UV-C) germicidal irradiation (to inactivate airborne microorganisms by damage to the DNA and prevent their ability to replicate), in addition to less common and emerging methods, such as hydrogen peroxide vapour, photocatalytic oxidation, and air ionisation [22,23,24,25].

A number of empirical studies on disinfection methods for airborne bacteria have been performed. However, the technical and fragmented nature of the published research, across engineering, infection control, microbiological, and clinical journals, has been identified as a barrier to greater awareness and uptake of indoor air disinfection methods by those with the responsibility for implementing them (e.g., infection control practitioners) [26]. Moreover, previous literature reviews on indoor air disinfection [22,27,28,29,30,31,32] have been largely narrative in nature, focused on a limited range of disinfection techniques, or restricted to a particular pathogen. Here, we sought to (1) systematically identify peer-reviewed studies of airborne bacterial respiratory pathogens, (2) perform quality assessment, (3) summarise their collective findings, and (4) highlight areas for future research.

## 2. Methods

### 2.1. Scope and Definitions

We conducted a systematic literature review in accordance with the Preferred Reporting Items for Systematic Review and Meta-analysis (PRISMA) guidelines [33]. Our review focused primarily on laboratory studies of indoor air disinfection methods, which focused on their performance for airborne bacteria under controlled conditions. We restricted our focus to studies of bacterial respiratory pathogens.

### 2.2. Search Strategy

We searched three databases: PubMed, Web of Science, and Scopus. One of the authors (Thi Tham Nguyen, T.T.N.) led the search, in consultation with an experienced research librarian, and another of the authors with subject matter expertise (Luke D. Knibbs, L.D.K.). In PubMed, both MeSH (Medical Subject Headings) terms and free-text terms related to indoor airborne transmission and air disinfection techniques were used. For consistency, the search terms were mapped as closely as possible to the other databases, in consultation with the librarian. No restriction was imposed on the earliest or latest year of publication. The search was restricted to original studies published in English-language journals up to 15 October 2020. The full search terms are provided (Table S1). 

We initially searched for a broader range of recent studies, including field studies of air disinfection focused on performance in ‘real-world’ settings and epidemiological studies, but we identified few contemporary studies meeting our criteria (five epidemiological studies [34,35,36,37,38], of which three dealt with human infection [36,37,38], and one non-human field study was done in a swine containment setting [39]).

### 2.3. Article Selection

After removing duplicates, the first author (T.T.N.) screened abstracts and titles for relevance. The full text of articles that passed screening was downloaded for further assessment against the inclusion criteria. If relevance could not be clearly determined from the abstract, the full text was downloaded. T.T.N. assessed each article’s eligibility against the inclusion criteria; any articles with unclear eligibility were referred to L.D.K. for assessment. Where the clinical relevance of a respiratory pathogen was unclear, a third reviewer with clinical expertise (Scott C. Bell, S.C.B.) determined if an article should be included.

### 2.4. Inclusion Criteria

Our inclusion criteria for studies were: (1) they reported numerical results for one or more airborne pathogenic bacteria, introduced by deliberate generation of bacteria under controlled conditions (typically nebulization into a controlled test chamber or mock room); (2) they reported the effects of one or more air disinfection methods; (3) the nature and level (where relevant) of the disinfection method were clearly stated; and, (4) the outcomes were quantitative measurements comparing the effect of the disinfection method to some reference condition (e.g., change in colony-forming units (CFUs), pathogen inactivation or capture rates, etc.). For example, the susceptibility of microorganisms to UV-C irradiation can be expressed as a Z-value, which describes the relationship between UV dose and the natural logarithm of colony counts of surviving organisms over time, and it is calculated as Z = (ln(N_0_/N_UV_)/D), where N_0_ is the colony count without UV-C exposure, Nuv is the colony count with UV-C exposure, and D is the UV-C dose [40]. Higher Z-values indicate greater susceptibility to UV-C.

### 2.5. Data Extraction

For studies meeting the inclusion criteria, we extracted information on the test setup and conditions (e.g., mock room, chamber), the nature of the disinfection method(s) and comparator condition(s), the microorganism(s) tested, nebulization method(s), sampling and microbiological method(s), airborne concentration(s), quantitative results on the effectiveness of the method(s), and the main conclusion(s).

### 2.6. Quality Assessment

We developed assessment tools to evaluate the quality of the studies. These were informed by the Newcastle–Ottawa scale [41], which is a standard tool for assessing the quality of epidemiological studies. The scale assigns a score (stars) to seven criteria, based on methodological quality, to provide an indicator of the overall quality of studies. We adapted the general framework of the scale to make it relevant to laboratory studies of airborne disinfection techniques. Details are in the supplement (S15–S26). Briefly, stars were allocated based on study design (maximum of 2 stars), methodological rigor (maximum of 5 stars), and presentation of results (maximum of 2 stars). A maximum of 9 stars could be given to each study, with scores of 0–5 stars indicative of lower quality studies, and scores of 6 or greater indicative of adequate quality studies. Only the adequate quality and above studies were included in this review.

## 3. Results

**Figure 1:**
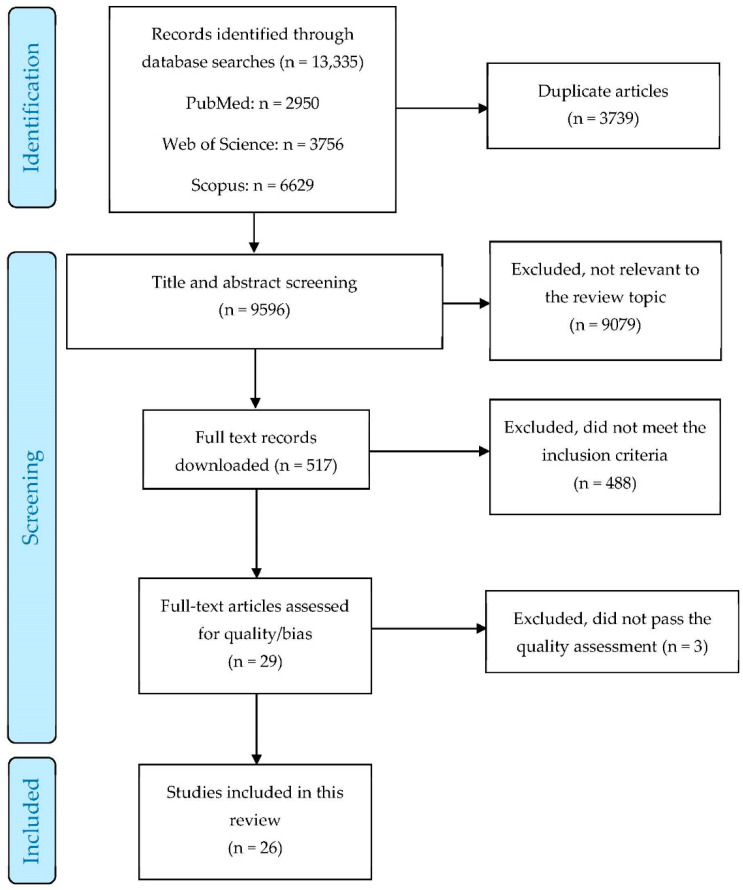
PRISMA flowchart presenting the search results and selection process of articles.

Figure 1 shows a PRISMA flowchart of the search and selection processes. After removing duplicates, 9596 titles and abstracts were screened. Of those, 9079 were excluded because they were not relevant. The full texts of the remaining 517 articles, which included articles with unclear relevance based on the abstract, were downloaded for further assessment. In total, 488 of those articles were excluded due to not meeting the inclusion criteria, resulting in 29 for further assessment. Finally, three articles did not meet the quality assessment threshold, providing 26 articles that were included in the systematic review. 

The details of the included studies and results of data extraction are presented in the supplement (Table S2). Briefly, a total of seven disinfection methods were identified across the 26 articles, including UV-C, filtration, masks, photocatalytic oxidation, electrostatic fields, cold plasma, and nanotechnology-based techniques with six studies utilising more than one method. Of the respiratory bacterial species investigated (total = 13), the majority were Gram-negative bacteria (*n* = 8, 62%), with fewer Gram-positive bacteria (*n* = 4, 31%), and one that was neither Gram-positive nor Gram-negative (8%, *M. tuberculosis*).

### 3.1. Filtration

Three studies assessed the effects of air filtration, of which two assessed a range of HVAC filters, while one study assessed an in-room filtration method. One of the studies focused on Gram-negative bacteria and two focused on both Gram-positive and Gram-negative type bacteria.

Commercially available air filters were reported to reduce airborne *Staphylococcus aureus* by 98.6 to 99.9% [42] and *Serratia marcescens* [43] by up 91% compared with no filtration. High-efficiency particulate air (HEPA) filters used in a mobile air-decontamination unit, coupled with a non-thermal reactor system, provided a single-pass 5-log reduction for airborne *Mycobacterium bovis* Bacillus Calmette-Guérin (BCG) and a 3-log reduction for *S. marcescens* [44].

### 3.2. UV-C (Wavelength 200–280 nm)

We identified 17 studies of UV-C, of which four assessed combined effects of UV-C and other methods (e.g., photocatalytic oxidation, filtration), six determined UV-C effectiveness in a chamber, four assessed upper-room UV-C installations, and three assessed in-duct UV-C. Of the 17 studies, seven focused on Gram-negative bacteria, five focused on Gram-positive bacteria, and five focused on both Gram-positive and Gram-negative bacteria. Overall, all studies demonstrated that the UV-C reduced viable airborne respiratory bacteria compared with comparator conditions, although the extent varied markedly depending on the specific organism and experimental conditions (Table 1).

Microorganism susceptibility to UV-C has been previously documented to vary due to the variation of the biological structure between bacteria, environmental exposure conditions (e.g., relative humidity (RH), temperature) and particle size distribution, among others [31,45]. For example, for a given UV-C dose (i.e., UV-C intensity [µW/cm^2^] × time [s]) under the same environmental conditions, *Pseudomonas aeruginosa* was reported to be more susceptible than *Legionella pneumophila* and *Staphylococcus aureus* [46].

Z-values for *M. tuberculosis*, *M. bovis* BCG, *S. marcescens*, and *Pseudomonas alcaligens* spanned two orders of magnitude, from 2 to 214 × 10^4^ cm^2^/μW s. Among those bacteria, *M. bovis* BCG was more resistant to UV-C, while *S. marcescens* and *P. alcaligens* were comparatively sensitive to UV-C [47,48,49,50,51,52]. There was generally an inverse relationship between RH and UV-C effectiveness. In the studies reviewed, Z values tended to decrease when RH increased, or bacterial susceptibilities at low RH were greater than those at higher RH [46,47,48,49,50,52]. However, one study showed an increase in inactivation rate when RH increased [53], and one recent study found no effect of RH on UV-C effectiveness [54].

**Table 1:** Z-values of UV-C effects on respiratory bacteria at different relative humidity levels.

Study	Microorganism	Relative Humidity (%)	Susceptibility Z × 10^4^ Value (cm^2^/µW s) (Range)
Riley et al., 1976 [47]	*Mycobacterium tuberculosis*—Erdman strain	50	33 (23–42)
	*Mycobacterium tuberculosis*—199RB strain	50	48 (44–55)
	*Mycobacterium bovis* BCG—culture 1	50	37 (33–39)
	*Mycobacterium bovis* BCG—culture 2	50	25 (23–28)
	*Mycobacterium bovis* BCG—culture 1	65	31
	*Mycobacterium bovis* BCG—culture 2	65	24
	*Serratia marcescens*	65	214 (183–245)
Ko et al., 2000 [48]	*Serratia marcescens*	22–33	58
	*Serratia marcescens*	49–62	57
	*Serratia marcescens*	85–91	−4
	*Mycobacterium bovis* BCG	22–33	27
	*Mycobacterium bovis* BCG	49–62	17
	*Mycobacterium bovis* BCG	85–91	2
Peccia et al., 2001 [49]	*Serratia marcescens*	40–50	35–45
Peccia et al., 2004 [50]	*Mycobacterium bovis* BCG	50	19.1
	*Mycobacterium bovis* BCG	95	~10 *
Yang et al., 2018 [51]	*Serratia marcescens*	55	120
	*Pseudomonas alcaligenes*	55	100
Zhang et al., 2019 [52]	*Pseudomonas alcaligenes*	50	85
	*Pseudomonas alcaligenes*	70	61
	*Pseudomonas alcaligenes*	90	34

* No exact value provided; this value has been estimated from the published figure in [50].

In the last two years, some studies have reported the use of upper-room UV-C systems with light-emitting diode (LED) lamps as opposed to more traditional mercury vapour discharge lamps. Lamps operated at 25%, 50%, and 100%, irradiance intensity output for 13 min resulted in 3.7, 5, and 6.4-log reductions for *S. marcescens,* respectively [55].

The number of air changes per hour (ACH) also affects upper-room and in-duct Upper-Room Ultraviolet Germicidal Irradiation (UVGI), because they, along with other factors, affect how long microorganisms are in the irradiated zone and whether they will be subject to repeated UV-C or one-off irradiation. For example, Ko et al. found that UV-C effectiveness increased by 7% (without a mixing fan) and by 24% (with a mixing fan) when ACH increased from 2 to 6 for *S. marcescens* [56]. When aerosolized bacteria were constantly generated for 90 min and the UVGI lamp was operated at full capacity (216 W), the concentration of airborne *M. Bovis* BCG in the breathing zone was reduced between 96 and 97% at 0 ACH [57]. Additionally, a portable UV-C (10 W UV lamp) apparatus inactivated >92% of airborne *S. marcescens* passing through the apparatus at an airflow rate of at least 50 cfm [58].

Few studies have assessed the combination of UV-C and other disinfection methods. When HEPA filtration was combined with UV, the system reduced more than 99.9% of viable airborne *Klebsiella pneumoniae*, *Acinetobacter baumannii*, and *S. aureus* after 45 min of operation in an aerobiology chamber [59,60]. An ultraviolet photocatalytic oxidation (UV-PCO) scrubber showed 3.5 to 5.3 log reduction of airborne *Enterococcus faecalis* with one UV lamp operating (average UV irradiation 6595 μW/cm^2^ at a contact time of 1 s) and a 7.5 log reduction with two lamps operating, although it should be noted that the irradiation varied markedly depending on proximity. The reduction efficiency of the scrubber was greater than 99.7% [61]. Finally, adding UV-C (intensity 1100 μW/cm^2^ to a commercial air filter increased the reduction of airborne *S. aureus* from about 67% to 99.7% [54].

### 3.3. Surgical Masks and Respirators

We identified three studies of masks or respirators. All of them assessed both surgical masks and N95 respirator masks. One study measured outward protection and two studies assessed inward protection. Among those studies, one focused on Gram-negative bacteria and two focused on Gram-positive bacteria.

N95 respirator masks and surgical masks were more effective in reducing cough-generated airborne *P. aeruginosa* release from people with cystic fibrosis (CF). Both surgical and N95 masks captured up to 94% of airborne *P. aeruginosa*, while cough etiquette (i.e., covering the mouth with a hand when coughing) captured up to 53% compared with uncovered coughing [62]. That study also reported significantly greater self-rated comfort for surgical compared with N95 masks.

In a study of *Bacillus anthracis*, the relative efficiency (i.e., the percent reduction of a test manikin’s inspired concentration of test aerosol when wearing the device compared to not wearing the device) of surgical and N95 masks ranged from 34 to 65% [63]. In a similar study, the relative efficiency of surgical masks and surgical N95 respirator masks ranged from 34 to 67% and 34 to 62% respectively, while N95 respirator masks had relative efficiency of 66 to 69% [64].

### 3.4. Other Air Disinfection Techniques

We identified three studies of other disinfection techniques. All of the studies focused on Gram-negative bacteria.

Several other techniques, such as engineered water nanostructure generated via electrospray (EWNS), non-thermal plasma-based technologies, and electrostatic fields [65,66,67], were examined; however, their performance varied. For example, a novel, chemical-free, nanotechnology-based method reduced by 50% airborne *S. marcescens* under steady-state conditions compared with the control without EWNS [65]. Cold plasma showed no reduction on airborne *S. marcescens* [65] and a reduction of <70% for airborne *P. alcaligenes* [66]. No significant reduction of airborne *Pseudomonas flourescens* by electrostatic fields was found when compared with the control condition [67].

### 3.5. Room Ventilation

No studies focused on room ventilation that met our inclusion criteria were identified.

## 4. Discussion

We systematically identified 26 largely laboratory-based studies of artificially generated aerosols, reporting the efficacy of different air disinfection methods to limit indoor airborne transmission of bacterial respiratory pathogens. It is not possible to directly compare the performance of these methods due to the relatively small number of studies and between-study methodological heterogeneity. Taken together, >80% of the studies suggest that air disinfection techniques were more effective at inactivating airborne bacteria than the comparator or baseline condition. However, this broad finding needs to be interpreted cautiously due to (a) the high proportion of studies on UV-C (17/26), (b) the variation in performance of all methods under different scenarios (i.e., how the disinfection method is implemented and which bacterial pathogen and strain it targets) and the indoor conditions (e.g., temperature, RH, particle size distribution, ventilation, room layout, movement of people).

The extent to which these studies reflect performance in ‘real-world’ indoor conditions varies, and it is not possible to speculate regarding the nature of this variation, because it will be dependent on the complex group of aforementioned factors. Given the small number of studies we identified, we suggest that there is a clear need for more, well-performed studies of air disinfection for bacterial pathogens. There is also a need for growing the evidence base on how the performance reported in laboratory studies relates to infection in human or animal model epidemiological studies. Therefore, the following section draws on other relevant literature beyond the studies reviewed here.

### 4.1. Evidence from Epidemiological Studies

Implementing 100% non-recirculated air and other droplet nuclei control measures in the trauma area of hospital reduced rates of tuberculin conversion of emergency department staff from 12% to 0% [37]. Menzies et al. undertook a cross-sectional study and found that low air exchange rates in non-isolation hospital rooms were robustly associated with tuberculin conversion among health care workers (hazard ratio: 3.4, 95% CI: 2.1–5.8 for rooms <2 ACH compared with ≥2 ACH) [38]. The same was not observed for isolation rooms [38]. An improvement in ventilation that reduced CO_2_ to less than 1000 ppm in university buildings was associated with a 97% decrease in the incidence of tuberculosis (TB) cases among contacts and a 38% decrease in the likelihood of latent TB infection among contacts [36].

Upper-room UVGI and negative ionization have been studied in guinea pig studies of TB transmission. Exhaust air from the rooms of HIV-positive patients with pulmonary TB was passed through guinea pig enclosures corresponding to different groups: ‘UV’, ‘ionizer’, and ‘control’. Thirty-five percent of the guinea pigs in the control group developed TB infection, while only 14% in the ionizer group and 9.5% in the UV group developed TB infection. This translated to 8.6%, 4.3%, and 3.6% with confirmed TB disease in these groups, respectively, which was statistically significant. A time-to-event analysis showed that both UV-C and ionizers were significantly associated with reduction in TB disease compared with control conditions, but UV-C was more effective than ionization [34].

Surgical face masks on patients with multidrug-resistant tuberculosis (MDR-TB) significantly reduced the transmission of airborne TB. A study in South Africa showed that 76.6% of guinea pigs in a control group (received air exhausted from MDR-TB patients without face masks) were infected with TB, while the TB infection rate of the intervention group (received air exhausted from MDR-TB patients with face masks) was 40% [35].

After the pioneering initial series of studies by Riley et al. of TB infection in guinea pigs in the 1950s and 1960 [68], remarkably few epidemiological studies of air disinfection for bacterial pathogens have been conducted, to our knowledge. Overall, they suggest benefits of air disinfection for reducing TB infection in human and animal-model studies; little is known about other respiratory bacterial pathogens. These knowledge gaps serve as a basis to motivate further fundamental laboratory studies, with a view to the scaling-up of promising methods in well-designed intervention trials focused on a wider range of pathogens.

### 4.2. Other Limitations of the Literature

Ultimately, only pathogens released from humans indoors reflect realistic conditions and survivability. Media suspensions for microorganism in lab-based studies may also affect their survivability in the air and their sensitivity to interventions [69]. For example, phosphate-buffered saline and artificial saliva conferred greater UV-C protection of airborne *S. marcescens* compared with nebulising using water or serum, at low RH conditions [69]. Differences between the compositions of the carrier fluid used to nebulise the organisms and the composition of naturally generated pathogen-laden aerosols may affect the representativeness of results. In addition, laboratory reference strains may not be fully representative of real-world pathogens, since they could have lost some of their physiological characteristics [70]. Clinical strains associated with disease might survive better in the air than other non-pathogenic strains [71]. Many studies have focused on non-pathogenic airborne microorganisms. These were deliberately excluded from our review. They are useful for demonstrating initial proof-of-concept of disinfection methods, but they have less clinical and public health significance.

### 4.3. Limitations of This Review

(1) This paper provides a qualitative systematic review; we did not undertake a meta-analysis or other quantitative assessment. We found the study design to be highly heterogeneous among the papers we reviewed. This highlights a need for greater standardisation of study design and testing protocols, where possible. (2) We only assessed respiratory pathogenic bacteria and did not attempt to assess the vast number of new publications on COVID-19, in the rapidly evolving landscape of evidence, and it was outside the scope of our review. (3) The infectious inoculum is unknown for many of the respiratory pathogens that we assessed, which makes it difficult to put the findings in context of human infection [72]. However, the epidemiological evidence we described provides some clues to suggest that air disinfection may reduce TB infection in human and animal-model studies. (4) Only English language papers were included in this systematic review for practical reasons. We do not believe our main findings would differ had our review extended to papers in other languages.

## 5. Conclusions and Suggestions

The literature on indoor air disinfection for respiratory bacteria is quite scarce, with only 26 studies identified that met our criteria. More than 80% of studies suggest that air disinfection techniques were more effective at inactivating airborne bacteria than the comparator or baseline condition, although the absolute reductions may not be relevant to human infection.

The lack of studies specific to room ventilation that met our inclusion criteria was surprising. This highlights that even for the disinfection method that has been known since the mid-19th century, and which can have beneficial effects on total bioaerosol levels in hospitals [73], further empirical studies specific to respiratory bacteria are clearly needed.

There are clear practical advantages to doing studies in controlled settings with nebulised bacteria, but in isolation, their individual or collective findings are less likely to be useful for infection control policy or procedures. Greater standardisation of experimental methods would make it easier to assess the body of literature as a whole in future.

Future research should focus more on testing methods that can be expanded into carefully designed epidemiological studies of human infection, particularly for non-TB pathogens. This may include methods that use a combination of different techniques (e.g., UV-C and filtration). Multidisciplinary study teams are needed for this work, as it does not sit neatly within any one field, requiring content expertise in medicine, microbiology, aerosol science, building design, engineering, and also engagement with the manufacturers of disinfection methods.

Although most studies we found were recent, some studies we included dated back to the 1970s, and progress in this field has been more sporadic than might be expected. It is possible that the COVID-19 pandemic may help to reinvigorate interest in airborne transmission control and spur new innovations that are relevant to bacterial pathogens.

## Data Availability

Not applicable.

## Supplementary Materials

The following supporting information can be downloaded at: https://www.mdpi.com/article/10.3390/ijerph19031197/s1, Table S1: Search strategies, Table S2: Data extraction, pages S15–S25: Quality assessment, pages S25–S26: Studies excluded due to not passing the quality assessment.
