# Supplementary material for: A Systematic Literature Review of Indoor Air Disinfection Techniques for Airborne Bacterial Respiratory Pathogens
Source: Int J Environ Res Public Health. 2022 Jan 21;19(3):1197. doi: 10.3390/ijerph19031197 (PMC8834760; doi:10.3390/ijerph19031197)
Supplement: Supplementary file 1 [file ijerph-19-01197-s001.zip › ijerph-1518272-supplementary.pdf]

SUPPORTING INFORMATION

Table S1: Search strategies

Web of Science

#1<br>Airborne transmission	(TS=(“airborne” OR “droplet*” OR “droplet nuclei” OR "airborne transmission" OR "air-borne transmission" OR "airborne infection*" OR "air-borne infection*"OR "airborne infection control*" OR “bioaerosol*” OR “bio-aerosol*” OR "air transmission" OR "droplet transmission" OR "infectious aerosol" OR "aerosol transmission")) AND **LANGUAGE:** (English)
#2<br>Air disinfection technologies	(TS= ("no-touch-techniq*" OR "no-touch disinfection" OR "UV" OR "UVC" OR "UV-C" OR "UVGI" OR "Ultraviolet germicidal irradiation" OR "UV light" OR "UV radiation" OR "ultraviolet" OR "ultra-violet" OR "ultra-violet rays" OR "ultraviolet rays" OR "ultra violet rays" OR "ultraviolet ray" OR "ultra-violet ray" OR "ultra violet ray" OR "photo-catalytic" OR "photocatalytic" OR "hydrogen peroxide" OR "superoxide" OR "superoxides" OR "hydroxyls" OR "hydroxyl" OR "hydroxyl radical" OR "air-ionisation" OR "air-ionization" OR "air ionization" OR "air ionisation" OR "ventilation" OR "HVAC" OR "isolation" OR "AII" OR "airborne infection isolation room" OR "airborne infection isolation rooms" OR "patient isolation" OR "hospital isolation" OR "pressure room" OR "negative pressure room" OR "HEPA" OR "HEPA filter" OR "filtration" OR "mask*" OR "surgical-mask*" OR "face-mask*" OR "face shield*" OR "face-shield*" OR "PPE" OR "personal protective equipment" OR "natural ventilation" OR "sunlight" OR "air disinfection" OR "air cleaning" OR "aerosol disinfection" OR "aerosol infection control*" OR "infection control method" OR "respiratory protective devices utilization" OR "air microbiology prevention and control")) AND **LANGUAGE:** (English)
#3<br>Method/Study design	(TS= ("laboratory" OR "chamber" OR "drum" OR "tunnel" OR "sampler" OR "aerosoliz*" OR "nebuliz*" OR "indoor" OR "indoor environment" OR "hospital" OR "clinical setting*" OR "healthcare setting*" OR "health care setting*" OR "office" OR "home" OR "domestic" OR "classroom" OR "school" OR "epidemiolog*" OR "epidemiologic* studies" OR "epidemiologic* study" OR "intervention study" OR "intervention studies" OR "cohort study" OR "cohort studies" OR "case-control studies" OR "case-control study" OR "case control study" OR "case control studies" OR "cross-sectional studies" OR "cross-sectional study" OR "longitudinal study" OR "longitudinal studies" OR "prospective studies" OR "prospective study" OR "retrospective studies" OR "retrospective study" OR "follow-up studies" OR "follow-up study" OR "before-after studies" OR "before-after study" OR "before and after studies" OR 'before and after study" OR "controlled before-after studies" OR "controlled before-after study" OR 'historically controlled studies" OR "historically controlled study" OR "interrupted time series analysis" OR "seroepidemiologic studies" OR "seroepidemiologic study" OR "clinical study" OR "clinical studies" OR "clinical trial" OR "clinical trials" OR "health surveys" OR "health survey" OR "public health surveillance" OR "controlled trial" OR "controlled trials" OR "incidence" OR "incidence rate" OR "hazard rate" OR "prevalence" OR "odds" OR "odds ratio" OR "relative risk" OR "risk ratio" OR "attributable risk" OR "infection rate" OR "inactivation rate" OR "transmission rate" R "hospital-acquired infections" OR "cross infection" OR "acute respiratory illness" OR "respiratory symptoms" OR "febrile acute respiratory disease" OR "febrile acute respiratory diseases" OR "pneumococcal infections" OR "nosocomial infections" OR "healthcare facilities" OR "health care facilities")) AND **LANGUAGE:** (English)
Strategy	(#1 AND #2 AND #3) AND **LANGUAGE:** (English)<br>*Indexes=SCI-EXPANDED, SSCI, A&HCI, CPCI-S, CPCI-SSH, BKCI-S, BKCI-SSH, ESCI, CCR-EXPANDED, IC Timespan=All years*

Scopus

#1<br>Airborne transmission	TITLE-ABS-KEY (airborne) OR TITLE-ABS-KEY (droplet*) OR TITLE-ABS-KEY ("droplet nuclei") OR TITLE-ABS-KEY ("airborne transmission") OR TITLE-ABS-KEY ("air-borne transmission") OR TITLE-ABS-KEY ("airborne infection*") OR TITLE-ABS-KEY ("air-borne infection*") OR TITLE-ABS-KEY ("airborne infection control*") OR TITLE-ABS-KEY (bioaerosol*) OR TITLE-ABS-KEY (bio-aerosol*) OR TITLE-ABS-KEY ("air transmission") OR TITLE-ABS-KEY ("droplet transmission") OR TITLE-ABS-KEY ("infectious aerosol") OR TITLE-ABS-KEY ("aerosol transmission")
#2<br>Air disinfection technologies	TITLE-ABS-KEY (no-touch-techniq*) OR TITLE-ABS-KEY ("no-touch disinfection") OR TITLE-ABS-KEY (uv) OR TITLE-ABS-KEY (uvc) OR TITLE-ABS-KEY (uv-c) OR TITLE-ABS-KEY (uvgi) OR TITLE-ABS-KEY ("ultraviolet germicidal irradiation") OR TITLE-ABS-KEY ("UV light") OR TITLE-ABS-KEY ("UV radiation") OR TITLE-ABS-KEY (ultraviolet) OR TITLE-ABS-KEY (ultra-violet) OR TITLE-ABS-KEY ("ultra-violet rays") OR TITLE-ABS-KEY ("ultraviolet rays") OR TITLE-ABS-KEY ("ultra violet rays") OR TITLE-ABS-KEY ("ultraviolet ray") OR TITLE-ABS-KEY ("ultra-violet ray") OR TITLE-ABS-KEY ("ultra violet ray") OR TITLE-ABS-KEY (photo-catalytic) OR TITLE-ABS-KEY (photocatalytic) OR TITLE-ABS-KEY ("hydrogen peroxide") OR TITLE-ABS-KEY (superoxide) OR TITLE-ABS-KEY (superoxides) OR TITLE-ABS-KEY (hydroxyls) OR TITLE-ABS-KEY (hydroxyl) OR TITLE-ABS-KEY ("hydroxyl radical") OR TITLE-ABS-KEY ("air-ionisation") OR TITLE-ABS-KEY ("air-ionization") OR TITLE-ABS-KEY ("air ionisation") OR TITLE-ABS-KEY ("air ionization") OR TITLE-ABS-KEY (ventilation) OR TITLE-ABS-KEY (hvac) OR TITLE-ABS-KEY (isolation) OR TITLE-ABS-KEY (aii) OR TITLE-ABS-KEY ("airborne infection isolation room") OR TITLE-ABS-KEY ("airborne infection isolation rooms") OR TITLE-ABS-KEY ("patient isolation") OR TITLE-ABS-KEY ("hospital isolation") OR TITLE-ABS-KEY ("pressure room") OR TITLE-ABS-KEY ("negative pressure room") OR TITLE-ABS-KEY ("positive pressure room") OR TITLE-ABS-KEY (hepa) OR TITLE-ABS-KEY ("HEPA filter") OR TITLE-ABS-KEY (filtration) OR TITLE-ABS-KEY (mask*) OR TITLE-ABS-KEY (surgical-mask*) OR TITLE-ABS-KEY (face-mask*) OR TITLE-ABS-KEY (face-shield*) OR TITLE-ABS-KEY ("face shield*") OR TITLE-ABS-KEY (ppe) OR TITLE-ABS-KEY ("personal protective equipment") OR TITLE-ABS-KEY ("natural ventilation") OR TITLE-ABS-KEY (sunlight) OR TITLE-ABS-KEY ("air disinfection") OR TITLE-ABS-KEY ("air cleaning") OR TITLE-ABS-KEY ("air microbiology prevention and control") OR TITLE-ABS-KEY ("aerosol disinfection") OR TITLE-ABS-KEY ("aerosol infection control") OR TITLE-ABS-KEY ("infection control method") OR TITLE-ABS-KEY ("respiratory protective devices utilization")
#3<br>Method/Study design	TITLE-ABS-KEY (laboratory) OR TITLE-ABS-KEY (chamber) OR TITLE-ABS-KEY (drum) OR TITLE-ABS-KEY (tunnel) OR TITLE-ABS-KEY (sampler) OR TITLE-ABS-KEY (aerosoliz*) OR TITLE-ABS-KEY (nebuliz*) OR TITLE-ABS-KEY (indoor) OR TITLE-ABS-KEY ("indoor environment") OR TITLE-ABS-KEY (hospital) OR TITLE-ABS-KEY ("clinical setting*") OR TITLE-ABS-KEY ("healthcare setting*") OR TITLE-ABS-KEY ("health care setting*") OR TITLE-ABS-KEY (office) OR TITLE-ABS-KEY (home) OR TITLE-ABS-KEY (domestic) OR TITLE-ABS-KEY (classroom) OR TITLE-ABS-KEY (school) OR TITLE-ABS-KEY (epidemiolog*) OR TITLE-ABS-KEY ("epidemiologic* studies") OR TITLE-ABS-KEY ("epidemiologic* study") OR TITLE-ABS-KEY ("intervention study") OR TITLE-ABS-KEY ("intervention studies") OR TITLE-ABS-KEY ("cohort study") OR TITLE-ABS-KEY ("cohort studies") OR TITLE-ABS-KEY ("case-control study") OR TITLE-ABS-KEY ("case-control studies") OR TITLE-ABS-KEY ("case control study") OR TITLE-ABS-KEY ("case control studies") OR TITLE-ABS-KEY ("cross-sectional study") OR TITLE-ABS-KEY ("cross-sectional studies") OR TITLE-ABS-KEY ("longitudinal study") OR TITLE-ABS-KEY ("longitudinal studies") OR TITLE-ABS-KEY ("prospective study") OR TITLE-ABS-KEY ("prospective studies") OR TITLE-ABS-KEY ("retrospective study") OR TITLE-ABS-KEY ("retrospective studies") OR TITLE-ABS-KEY ("follow-up study") OR TITLE-ABS-KEY ("follow-up studies") OR TITLE-ABS-KEY ("before-after study") OR TITLE-ABS-KEY ("before-after studies") OR TITLE-ABS-KEY ("before and after study") OR TITLE-ABS-KEY ("before and after studies") OR TITLE-ABS-KEY ("controlled

	before-after study") OR TITLE-ABS-KEY ("controlled before-after studies") OR TITLE-ABS-KEY ("historically controlled study") OR TITLE-ABS-KEY ("historically controlled studies") OR TITLE-ABS-KEY ("interrupted time series analysis") OR TITLE-ABS-KEY ("seroepidemiologic studies") OR TITLE-ABS-KEY ("seroepidemiologic study") OR TITLE-ABS-KEY ("clinical study") OR TITLE-ABS-KEY ("clinical studies") OR TITLE-ABS-KEY ("clinical trial") OR TITLE-ABS-KEY ("clinical trials") OR TITLE-ABS-KEY ("health surveys") OR TITLE-ABS-KEY ("health survey") OR TITLE-ABS-KEY ("public health surveillance") OR TITLE-ABS-KEY ("controlled trial") OR TITLE-ABS-KEY ("controlled trials") OR TITLE-ABS-KEY (incidence) OR TITLE-ABS-KEY ("incidence rate") OR TITLE-ABS-KEY ("hazard rate") OR TITLE-ABS-KEY (prevalence) OR TITLE-ABS-KEY (odds) OR TITLE-ABS-KEY ("odds ratio") OR TITLE-ABS-KEY ("relative risk") OR TITLE-ABS-KEY ("risk ratio") OR TITLE-ABS-KEY ("attributable risk") OR TITLE-ABS-KEY ("infection rate") OR TITLE-ABS-KEY ("inactivation rate") OR TITLE-ABS-KEY ("transmission rate") OR TITLE-ABS-KEY ("hospital-acquired infections") OR TITLE-ABS-KEY ("cross infection") OR TITLE-ABS-KEY ("acute respiratory illness") OR TITLE-ABS-KEY ("respiratory symptoms") OR TITLE-ABS-KEY ("febrile acute respiratory disease") OR TITLE-ABS-KEY ("febrile acute respiratory diseases") OR TITLE-ABS-KEY ("pneumococcal infections") OR TITLE-ABS-KEY ("nosocomial infections") OR TITLE-ABS-KEY ("healthcare facilities") OR TITLE-ABS-KEY ("health care facilities")
Strategy	(1 and #2 and #3) AND (LIMIT-TO (LANGUAGE , "English"))

Pubmed

#1 Airborne transmission	airborne[tiab] OR droplet*[tiab] OR "droplet nuclei"[tiab] OR "airborne transmission"[tiab] OR "air-borne transmission"[tiab] OR "air-borne infection"[tiab] OR "airborne infection control*"[tiab] OR bioaerosol*[tiab] OR bio-aerosol*[tiab] OR "air transmission"[tiab] OR “droplet transmission” OR “infectious aerosol”[tiab] OR “aerosol transmission”[tiab]
#2 Air disinfection technologies	no-touch-techniq*[tiab] OR "no-touch disinfection"[tiab] OR UV[tiab] OR UVC[tiab] OR UV-C[tiab] OR UVGI[tiab] OR "Ultraviolet germicidal irradiation"[tiab] OR "UV light"[tiab] OR "UV radiation"[tiab] OR ultraviolet[tiab] OR ultra-violet[tiab] OR "ultra-violet rays"[tiab] OR "ultraviolet rays"[tiab] OR "ultraviolet ray"[tiab] OR "ultra-violet ray"[tiab] OR "ultra violet ray"[tiab] OR "photo-catalytic"[tiab] OR "photocatalytic"[tiab] OR "hydrogen peroxide"[tiab] OR "superoxide"[tiab] OR "superoxides"[tiab] OR hydroxyl[tiab] OR hydroxyls[tiab] OR "hydroxyl radical"[tiab] OR air-ionization[tiab] OR air-ionisation[tiab] OR "air ionisation"[tiab] OR "air ionization"[tiab] OR ventilation[tiab] OR HVAC[tiab] OR isolation[tiab] OR AII[tiab] OR "airborne infection isolation room"[tiab] OR "airborne infection isolation rooms"[tiab] OR "hospital isolation"[tiab] OR "patient isolation"[tiab] OR "pressure room"[tiab] OR "negative pressure room"[tiab] OR HEPA[tiab] OR "HEPA filter"[tiab] OR filtration[tiab] OR mask*[tiab] OR surgical-mask*[tiab] OR face-mask*[tiab] OR face-shield*[tiab] OR "face shield*"[tiab] OR PPE[tiab] OR "personal protective equipment"[tiab] OR "natural ventilation"[tiab] OR sunlight[tiab] OR "air disinfection"[tiab] OR "air cleaning"[tiab] OR "Ultraviolet Rays"[Mesh] OR "Hydrogen Peroxide"[Mesh] OR "Superoxides"[Mesh] OR "Hydroxyl Radical"[Mesh] OR "Air Ionization"[Mesh] OR "Ventilation"[Mesh] OR "Hospitals, Isolation"[Mesh] OR "Patient Isolation"[Mesh] OR "Masks"[Mesh] OR "Personal Protective Equipment"[Mesh] OR "Sunlight"[Mesh:NoExp] OR “air disinfection”[tiab] OR “air cleaning”[tiab] OR “aerosol disinfection”[tiab] OR “aerosol infection control*”[tiab] OR “infection control*/method”[Mesh] OR “Respiratory protective devices/utilization”[Mesh] OR “air microbiology/prevention and control”[Mesh]
#3	aerosol OR laboratory OR chamber OR drum OR tunnel OR sampler OR aerosoliz* OR nebuliz* OR indoor OR "indoor environment" OR hospital OR "clinical setting*" OR "healthcare setting*" OR "health

Method/Study design	care setting*" OR office OR home OR domestic OR classroom OR school OR epidemiolog* OR "epidemiologic* studies" OR "epidemiologic* study" OR "intervention study" OR "intervention studies" OR "cohort studies" OR "cohort study" OR "case-control study" OR "case-control studies" OR "case control studies" OR "case control study" OR "cross-sectional study" OR "cross-sectional studies" OR "longitudinal study" OR "longitudinal studies" OR "prospective studies" OR "prospective study" OR "retrospective study" OR "retrospective studies" OR "follow-up studies" OR "follow-up study" OR "before-after study" OR "before-after studies" OR "before and after studies" OR "before and after study" OR "controlled before-after study" OR "controlled before-after studies" OR "historically controlled studies" OR "historically controlled study" OR "interrupted time series analysis" OR "seroepidemiologic studies" OR "seroepidemiologic study" OR "clinical study" OR "clinical studies" OR "clinical trial" OR "clinical trials" OR "health surveys" OR "health survey" OR "public health surveillance" OR "controlled trial" OR "controlled trials" OR incidence OR "incidence rate" OR "hazard rate" OR prevalence OR odds OR "odds ratio" OR "relative risk" OR "risk ratio" OR "attributable risk" OR "infection rate" OR "inactivation rate" OR "transmission rate" OR "hospital-acquired infections" OR "cross infection" OR "acute respiratory illness" OR "respiratory symptoms" OR "febrile acute respiratory disease" OR "febrile acute respiratory diseases" OR "pneumococcal infections" OR "nosocomial infections" OR "Laboratories, Hospital"[Mesh] OR "Hospitals"[Mesh] OR "Schools"[Mesh] OR "Epidemiology"[Mesh] OR "Epidemiologic Studies"[Mesh] OR "Clinical Study" [Publication Type] OR "Public Health Surveillance"[Mesh] OR "Incidence"[Mesh] OR "Prevalence"[Mesh] OR "Odds Ratio"[Mesh] OR "Cross Infection"[Mesh:NoExp] OR "Pneumococcal Infections"[Mesh:NoExp]
Strategy	1 AND #2 AND #3

Table S2: Data extraction

S/N	Paper (author, year)	Test location	Microorganisms	Initial concentration	Disinfection techniques	Nebulized techniques	Sampling methods	Microbiology	Results	Conclusion
1	Wenke C et al., 2017	Laboratory	Equine arteritis virus (EAV), Porcine reproductive and respiratory syndrome virus (PRRSV), Bovine enterovirus 1 (BEV), *Actinobacillus pleuropneumobiae* (APP), and *Staphylococcus aureus*	$10^{6.7}$–$10^{7.6}$ $TCID_{50}$/ml (EAV), $10^{5.3}$–$10^{5.8}$ $TCID_{50}$/ml (PRRSV), and $10^{5.3}$–$10^{5.6}$ $TCID_{50}$/ml (BEV) 10^{8}−10^{9} cfu/ml (*S. aureus*), 6×10^{8} cfu/ml (*APP*)	Four mechanical filters: prototypes 1 and 2 were composed of a prefilter and a secondary filter whereas prototypes 3 and 4 consisted of a filter wool mat. The secondary filters of both, prototype 1 and 2, had been determined to be > 95% efficient at removing particles equal to or greater than 0.4 µm in diameter.	Atomizer Aerosol generator ATM 230.	Air was collected using an air sampler pump and water-soluble gelatin filters, flowrate of 550 L/h for 20 min. Samples were collected in front of and behind the filter.	Bacteria were enumerated by the spread-plate method. Virus titration and quantitative real-time RT-PCR used for virus.	Bacteria were most efficiently filtered with a reduction rate of up to 99.9% (92.1-99.9%). An approximately 98% reduction was achieved for the viruses tested. Viability or infectivity of APP or PRRSV in the filter material decreased below the detection limit after 4 h and 24 h respectively, whereas *S. aureus* was still culturable after 4 weeks.	Air filtration combined with other biosecurity measures markedly reduces the risk of introducing airborne transmitted pathogens to animal facilities and might be useful in reducing bioaerosols within a pig barn, hence improving respiratory health of pigs.
2	Furuhashi M et al., 1978	Apparatus	*Staphylococcus aureus* and *Serratia marcescens*	2.2×10^{5} to 2×10^{6} cells/ml	HEPA filters, 3 kinds. NBS-95, NBS-85, NBS-75 air filters Activated carbon medium, synthetic and inorganic fiber air filter Activated carbon fiber mat air filter.	Chicago nebulizer, compressed air was at a pressure of 15 lb/in^{2}.	6-stage Andersen sampler, operated at a flowrate of 28.3 L/min.	Trypticase-soy agar (TSA) plates were incubated for 37 ± 1 °C for *S. aureus* and 27 ± 1 °C for *S. marcescens* for 24-48 h.	To *S. marcescens*, three types of HEPA filters showed 100% bacterial filtration efficiency (BFE), NBS-95 and NBS-85 filters showed over 99% BFE, NBS-75 showed 91.75%. BFE of two types of activated charcoal filters and K filters were 42.6 ± 10.7% and 66.5 ± 6.82% respectively.	HEPA filter materials were the most effective.

3	Bergeron V et al., 2011	Test room	H5N2 influenza virus, BCG *Mycobacterium bovis* and *S. marcescens*	20 mL of 1 × 10^7 $TCID_{50}$ for H5N2, 10^6 cfu/mL (BCG *M. bovis* and *S. marcescens*)	The mobile air-decontamination unit was equipped with HEPA filtration that achieved with single-pass particle removal efficiencies of >99.97% for particulate matter ≥ 0.3 µm. Besides, non-thermal plasma reactor system was to inactivate captured microorganisms.	6-jet Collison nebulizer, nebulized at 180 kPa for 30 min.	AGI 30 liquid impingers, air flow rate was 25 L/min, and sampled for 5 min.	10 mL fluid was collected in PBS (BCG and *S. marcescens*). BCG was inoculated onto Middlebrook 7H10 agar plates, incubated for 3 weeks at 37 °C and *S. marcescens* was inoculated onto plate count agar, incubated for 24, 48, and 72 h at 32 °C.	Single pass study: for *M. bovis*, a 5-log reduction was obtained when the system was on, compared with ~1-log reduction when the system was off. For *S. marcescens*, a 3-log reduction when the system was on an a 0.4-log reduction when the system was off.	High-throughput in-room air treatment units showed its effects in reducing airborne viruses and bacteria, thus can provide additional control of airborne pathogen levels in patient isolation rooms.
4	Sattar SA et al., 2016	Chamber	*Staphylococcus aureus* and *Klebsiella pneumoniae*	4.61 $\log_{10}$ cfu/m^3 for *S. aureus* and 4.62 $\log_{10}$ cfu/m^3 for *K. pneumoniae*	3 types of commercial air de-contamination devices equipped with 5 (device 1), 8 (device 2), and 9 (device 3) W UV light bulbs.	6-jet Collison nebulizer.	Slit-to-agar sampler STA, run at 28.3 L/min.	Plate culture: Modified Letheen agar for both bacteria, incubated at 36 °C ± 1 for 18 ± 2 hours. The plates with few or no colonies were incubated for a total of 5 days to allow any slow-growing stressed or injured bacteria to form colonies.	For both *S. aureus* and *K. pneumoniae*, devices 1 and 2 could achieve a ≥ 3 log10 reduction in bacterial viability in 45 minutes, with no recoverable CFU from the air in the chamber after 60 min, device 3 was less effective than the other devices.	The experimental setup used is also versatile enough to allow for testing against any types of microorganisms.
5	B. Zargar et al., 2018	Chamber	*Staphylococcus aureus, Acinobacter baumannii*; and a bacterial spore former (*Geobacillus stearothermophilus*)	From 4.4 $\log_{10}$ to 4.9 $\log_{10}$ cfu/m^3.	Commercial device Germ Guardian AC4825, based on a combination of HEPA filtration and UV irradiation.	Six-jet Collison nebulizer (25 psi, 172.4 kPa).	Surface-to-agar air sampler, operated at the rate of 28.3 L/min, collected air samples for 2 to 10 min.	*Staphylococcus aureus* and *A. baumanii* were grown aerobically in trypticase soy broth at 36 ± 1 °C for 18 ± 2 h. The spores of *G. stearothermophilus* was grown in the same media at 56 ± 1 °C.	The device inactivated >99.9% nebulized bacteria in 45 min of its operation.	The combination of HEPA filtration and UV irradiation worked in removing tested bacteria and might have potential for use with other types of bacterial airborne pathogens or fungi and viruses.
6	Eisenloffel L et al., 2019	Chamber	*Staphylococcus aureus, Actinobacillus pleuropneumoniae* (APP), porcine parvovirus (PPV) and porcine reproductive and respiratory syndrome virus (PRRSV).	Bacterial suspension: 10^8-10^9 cfu/ml; PRRSV: $10^{5.6}$ - $10^{6.1}$ $TCID_{50}$/ml; PPV: $10^{6.8}$ - 10^7 $TCID_{50}$/ml	Commercial air filters with and without UVGI within the filter.	Atomizer Aerosol generator ATM 230.	Water soluble gelatin filters.	Gelatin filters were dissolved in 5 ml of tryptic soy broth or cell culture medium at 37 °C. Spread-plate method was used for determining bacterial numbers and virus titration used for viruses.	UVC irradiation itself or in combination to air filter reduced > 99% bacteria and viruses whereas filter alone reduced 67.7% *S. aureus*.	Combination of UVC irradiance to air filtration was successful in reducing airborne bacteria at laboratory.

7	Li CS et al., 2003	Chamber	*Legionella pneumophila*	10^6 cfu/mL	UVGI: low-pressure mercury-vapor discharge UV-C germicidal lamps with the exposure time of 0.55 s.<br>Photoreactor: a commercial TiO_2 filter was selected as a photocatalyst that can be excited by photons with wavelength < 385 nm. The light intensity of the filter surface from 8-W lamps was 7.4 mW/cm^2.	Collison 6-jet nebulizer operated at 3 L/min and passed through a Kr-85 particle charge neutralizer.	Andersen 6-stage sampler, run at a flowrate of 28.3 L/min. AGI-30 all-glass impingers as a reference sampler, run at 12.5 L/min with for 5 min.	BCYE α agar, incubated in 5% CO_2 for 3 days at 37 °C.	A very low UV-C dose of 289 to 860 μW s/cm^2 was required to produce a 5 log decrease in concentrations of *L. pneumophila.*<br>Penetrations of *Legionella pneumophila* were from 0.04-0.27 using the TiO_2-coating filter with UV light.	UVGI and the TiO_2 catalyst filter had a good germicidal capacity for *L. pneumophila.*
8	King B et al., 2011	Chamber	*Bacillus atrophaeus*, *Pantoea agglomerans*, and *Yersinia ruckeri*	*B. atrophaeus*: 1 × 10^9 cfu/ml<br>*P. agglomerans*: 9 × 10^9 cfu/ml<br>*Yersinia ruckeri*: 3 × 10^9 cfu/ml	UV-C lamps, either 1 or two 15-W bulbs. The flux measured at the location of the dishes was 0.85 W/m^2 for the low UV-C setting (1 UV bulb) and 1.6 W/m^2 for the high UV-C setting (2 UV bulbs). UV-C doses ranged from 0 to 576 J/m^2.	24-jet Collison nebulizer, operated at 20 psi, aerosolized bacteria for 10 min.	3-μm pore size, 47-mm diameter gelatin filters with pump run at 12.5 L/min for *B. atrophaeus*, 50 L/min for the other bacteria. The sampling time was 1 min.	The plates incubated overnight at 37 °C (*B. atrophaeus* and *P. agglomerans*) or at room temperature (21 °C, *Y. ruckeri*).	The fluence of UVC required to inactivate 90% of aerosolized bacteria were 70.3 J/m^2, 73.3 J/m^2 and 18.3 J/m^2 for vegetative cells of *B. atrophaeus*, *P. agglomerans,* and *Y. ruckeri* cells respectively.	This study confirmed the effectiveness of UV-C on different bacterial species.
9	Riley RL et al., 1976	Test room	Bacille Calmette-Guerin (BCG), *Mycobacterium tuberculosis*, *Mycobacterium phlei*	*M. tuberculosis*: 1 × 10^4 viable units/ml<br>BCG: 1 × 10^6 viable units/ml<br>*M. phlei*: 4 × 10^3 viable units/ml	2 UV fixtures with the UV tubes operated at 17 W and 29 W. The bacteria were exposed for 6 s to 21 s at the intensity of 85 μW/cm^2. The UV dose was 126 (6 x 21) or 510 (6 x 85) μW s/cm^2.	Bird nebulizer at a line pressure of 15 psi.	6-stage Andersen sampler.	*M. tuberculosis* Middlebrook, *M. bovis*: Cohn 7H10 agar plate, incubated at 37 °C for 3 weeks and *M. phlei*: nutrient agar with 4% defibrinated sheep's blood, incubated at 37 °C for 4 days.	The Z values (cm^2/μW s) at RH 50% were 33 × 10^4, 48 × 10^4 for *M. tuberculosis* the Erdman and 199RB strain respectively. To *M. bovis*, culture 1 and 2 had Z value 37 × 10^4 and 25 × 10^4, the overall Z value for BGC was 31 × 10^4, for *M.plei* was 4 × 10^4.	Upper air UV-C irradiation was effective in against the resistant BCG and virulent tubercle bacilli.

10	Ko G et al., 2000	Chamber	*Serratia marcescens*, *Mycobacterium bovis* bacille Calmette-Guerin (BCG)	10^4-10^5 cfu/ml for both bacteria.	A UV-C fixture including six 36 W UV-C lamps installed above a fused quartz UV window in the main body of the chamber. The UV-C irradiance were 10.6, 36.6 and 77.4 $\mu W/cm^2$ and exposure time were 3.8 and 7.6 s.	Six-jet Collison nebulizer, operated at 138 kPa (20 psi).	Six-stage Andersen cascade impactor.	Nutrient agar was used for *S. marcescens* and Middlebrook 7H10 agar plates were used for BCG.<br>Culture plates were incubated at room temperature for 2-3 days for *S. marcescens* and for 3-4 weeks at 37°C for BCG.	Mean Z values of *S. marcenscens* were 58, 57 and 4 × 10^4 $cm^2/\mu W$ s at low (22-33), medium (49-62) and high (85-91) RH and those of BCG were 27, 17 and 2 $cm^2/\mu W$ s respectively. When RH increased, both bacteria were more resistant to UV-C, particularly when RH higher than 85%.	RH had a great effect on UV-C effectiveness in upper room air. RH inside the buildings should be kept at <75% to optimize the inactivation rate of UV-C.
11	Peccia J et al., 2001	Chamber	*Serratia marcescens*, *Bacillus subtilis*, and *Mycobacterium parafortuitum*	1 × 10^9 cfu/ml	30-W low-pressure, mercury vapor UV-C lamps, wavelength of 254 nm. Three 1.5 W fans ran continuously inside the chamber during the experiments. The average spherical irradiances measured in the chamber were 7.53 $\mu W/cm^2$ and 3.7 $\mu W/cm^2$ for four and two lamps operating respectively.	Collison 6-jet nebulizer, operated at 20 psi, aerosolized in 3 min.	Glass impingers (AGI-30), run at 12.5 L/min and 6-stage Andersen impactor, run at 28.3 L/min.	Soybean-Casein digest agar from impactor were incubated at 37 °C. Samples from liquid impingers were diluted and plates then for *S. marcescens* and *B. subtilis*: incubated at 37 °C for 24 h and for *M. parafortuitum*: incubated at 37 °C for 3 days.	Z value of *M. parafortuitum* was 12-15 × 10^4 $cm^2/\mu W$ s at 50% RH; Z value of *B. subtilis* was 6.3-6.6 × 10^4 $cm^2/\mu W$ s at RH from 20-40%; Z value of *S. marcescens* was 35-45 × 10^4 $cm^2/\mu W$ s at 40-50% RH.	UV-C effectiveness was dependent on RH. Inactivation rate of UV-C decreased sharply when RH exceeded 50%.
12	Peccia J et al., 2004	Bench-scale reactor	*Mycobacterium bovis* bacillus Calmette-Guerin (BCG)	1 × 10^6 cfu/ml	Low-pressure, 30 W, mercury vapor UV-C lamps were installed and were wrapped in 8 layers of aluminum filter mesh. The average UV-C spherical radiance in aerosol reactor was 7.53 ± 0.13 $\mu W/cm^2$. The reactor was mixed by three 1.5 W fans ~ ACH 0 to 7.3.	6-jet Collison nebulizer, run at 20 psi for 5 min.	Glass impingers (AGI-30).	7H11 agar plates were incubated at 37 °C for 21 days.	Z values of *M. bovis* BCG at 50% RH and 95% RH were 19.1 × 10^4 $cm^2/\mu W$ s and approximately 10 × 10^4 $cm^2/\mu W$ s respectively.	Relative humidity had effects on UV-C inactivation rate for *M. bovis* BCG. RH must be considered as a critical design parameter when using UV-C as an aerosol disinfection technique.

13	Yang Y et al., 2018	Test room	*Serratia marcescens*, *Pseudomonas alcaligenes*, *Escherichia coli*, *Salmonella enterica*, and *Staphylococcus epidermidis*	NA	200 × 200 mm ventilation duct UVGI system (in-duct UVGI). One 10 W UV-C lamp was inserted from sidewall of the duct.	24-jet Collison nebulizer, with an inlet pressure 275.8 kPa, generated bacteria for 15 min.	Single-stage Andersen cascade impactor, run at 28.3 L/min for 106 s.	During incubation, all types of bacteria were grown under a constant temperature of 37°C except for *P. alcaligenes* at 30°C.	By varying the UV intensity, the susceptibility constants (Z- values) of the bacteria were experimentally determined to be 1.2, 1.0, 0.60, 0.39, and 0.37 m^2/J for *S. marcescens*, *P. alcaligenes*, *E. coli*, *S. enterica*, and *S. epidermidis* respectively.<br>The disinfection efficacy decreased with increasing inlet velocity.	The in-duct UVGI system potentially provides an additional option for improving indoor air quality within mechanical ventilated/air-conditioned environment.
14	Zhang H et al., 2020	Laboratory	*Staphylococcus epidermidis, Pseudomonas alcaligenes,* and *Escherichia coli*	NA	200 × 200 mm ventilation duct UVGI system (in-duct UVGI). UV-C lamp was a low-pressure mercury-vapor lamp (9W) with twin tubes 10 mm in diameter and with a luminous length of 120 mm. The UV intensities of 2/3, 1/3, and 1/6 UV-C lamps at distance of 150 mm from the UV lamp under background laboratory condition were 370.5, 203.8, and 137.8 $\mu w/cm^2$ respectively.	24-jet Collison nebulizer, air pressure was maintained at 103.42 kPa (15 psi).	Single stage Andersen cascade impactor, pump operated at 28.3 L/min.	Agar plates were incubated at 37 °C for 24 h for *S. epidermidis* and *E. coli* and 48 h for *P. alcaligenes*.	At 50% RH, Z values of *S. epidermidis*, *P. alcaligenes*, and *E. coli* were 0.3, 0.85, and 0.5 m^2/J respectively. When RH increased from 50 to 90%, the Z values decreased by ~ 40, 60, and 38% for *S. epidermidis*, *P. alcaligenes*, and *E. coli* respectively.<br>Highest disinfection efficacy was at 20-21 °C, lower or higher temperature decreased the efficacy of UV-C.<br>Increased airflow velocity decreased UV-C efficacy.	Thorough understanding of environmental factors affecting to UV-C efficacy is useful for practical application of UV-C, and proper installment of UV lamps at the optimal locations.

15	Nunayon SS. et al., 2019	Test room	*Escherichia coli, Serratia marcescens,* and *Staphylococcus epidermis*	10^8 cfu/ml	Novel UR-UVGI-LED device: consisted of 10 UVC-LEDs, the total rated optical output for 10 UVC-LEDs was >200 mW, and when the total input current was 3.5A, the intensity was 40.77 μW/cm^2. 10 individual UVC-LEDs were arranged in the form of a well-packed rectangular array that was hung midway from one of the walls of the test room at a height of 2.01 m.<br><br>Conventional UR-UVGI-MV system: a 21 W modern UVGI lamp, installed on the north wall, opposite the door of the test room. Average UV irradiance was 18.95 μW/cm^2 at the irradiated zone.	24-jet Collison nebulizer, aerosolized bacteria for 5 min.	Single stage Andersen cascade impactor, pump operated at 28.3 L/min.	Nutrient agar (NA, BD) plates were incubated at 37 °C for 24 h.	For *S. marcescens*, UR-UVGI-LED operated at 25%, 50% and 100% irradiation intensity for 13 min, bacterial concentration reduced 3.7 $\log_{10}$, 5 $\log_{10}$ and 6.4 $\log_{10}$ respectively. UR-UVGI-MV system produced a 6.8 $\log_{10}$ reduction for *S. marcescen*.	UR-UVGI-LED system is promising tool for future use in airborne pathogen disinfection. It can be slightly more energy cost-effective compared with UR-UVGI-MV system.
16	Xu P et al., 2003	Test room	*Bacillus subtilis* (spores), *Mycobacterium parafortuitum*, and *Mycobacterium bovis* BCG cells	1 × 10^9 cfu/ml	Ventilation system 2 to 8 ACH, the room air was mixed with 2 box fans. UVGI system consists of 2 luminaries. The center luminary is rated at 72 W, consisting of 4 lamps. The corner luminaries included 2 lamps, rated at 36 W each.	Six-jet Collison nebulizer, run at 137 kPa (20 psi).	Glass impingers (AGI-30), run at 12 L/min for 30 min at 9 sampling locations.	Air samples were diluted and plated, then the plates were incubated at 37 °C for 24 h for *B. subtilis*, for 60 h for *M. parafortuitum*, and for 21-35 days for *M. bovis* BCG. All organisms were plated on SCDA agar except *M. bovis* BCG, which was plated on 7H11 media.	UVGI reduced the room-average concentration of culturable airborne bacteria between 46% and 80% for *B. subtilis* spores between 83% and 98% for *M. parafortuitum* depending on the ventilation rate, and 96–97% for *M. bovis* BCG cells at 0 ACH.<br><br>The Z value was 1.2 ± 0.15 x 10^3 cm^2/μW s for aerosolized *M. parafortuitum* at 50% RH.	A linear relationship was observed between the UVGI inactivation rate, ACH, and level of UV irradiance.

17	Ko G et al., 2002	Chamber	*Serratia marcescens* and *Mycobacterium bovis* BCG	*S. marcescens*: 1.5 × 10^6 cfu/ml<br>*M. bovis* BCG: 10^5 cfu/ml	The wall-mounted UV fixture contained one 23-W UV lamp (output 5 W) and the ceiling-mounted UV fixture contained four 9-W UV lamps (output 10 W).	6-jet Collison nebulizer, air pressure was at 138 kPa.	6-stage Andersen impactor, sampled for 4 min.	Nutrient agar for *S. marcescens* and Middlebook agar for *M. bovis* BCG. Plates were incubated at room temperature for 48 h for *S. marcescens* and at 37 °C for 3 weeks for BCG.	The use of ceiling- and wall-mounted UV fixtures without mixing fan reduced the concentration of *S. marcescens* aerosols by 46% at 2 ACH and 53% at 6 ACH. The use of ceiling and wall mounted UV fixtures with mixing fan increased the UV effectiveness to 62% at 2 ACH and to 86% at 6 ACH. UV effectiveness in inactivating BCG aerosols at 6 ACH were 52% by ceiling mounted UV fixture only and 64% by both ceiling- and wall mounted UV fixtures.	Upper room UVGI can significantly reduce the concentration of aerosolized microorganisms (*S. marcescens* and BCG). Both air exchange rate and mixing fan affected UV-C effectiveness.
18	Chang CW et al., 2013	Chamber	*Staphylococcus aureus*, *Pseudomonas aeruginosa*, and *Legionella pneu-mophila*	~ 9 $\log_{10}$ cfu/ml	Central UV-C lamp with UV-irradiance intensities were averaged as 12.6, 6.1, 3.8, and 2.9 mW/cm^2 respectively at 0-, 3-, 6-, and 9-cm distances from the UV lamp. The UV exposure time of bioaerosols was regulated by adjusting the Q passing through the UV unit. Q was adjusted at flow rate of 30 or 60 L/min.	Collison three-jet nebulizer, at 3 L/min.	AGI-30 glass impingers, pump run at 12.5 L/min, samplings were done for 10 and 30 min.	Two kinds of plating were conducted with 5 replicate plates each because of low CFU counts: (i) by mixing 1 ml of sample with 10 mL of melted TSA or BCYEα medium (45 ± 2°C) and (2) by spreading 0.2 mL of sample on TSA and BCYEα agar. After incubation in the dark at 37°C for 1 day (TSA) or 4 days with 5% CO2 (BCYEα), CFU counts from 5 replicate plates were summed up and divided by total sample volume analyzed.	The highest UVGI effectiveness against airborne *S. aureus* occurred at low RH (12.7–16.7%) regardless of sampling time, with a mean reduction of bioaerosol concentration by 4.8–4.9 and 3.9–4.4 log units at 30 and 60 L/min respectively.<br>The highest susceptibility of *P. aeruginosa* to UVGI was observed at low RH regardless of sampling time and Q, with a mean reduction of 4.5–4.9 and 4.4–4.5 log units at 30 and 60 L/min, respectively.<br>For *L. pneumophila,* log-reduction values at high RH (3.1–3.6 and 2.2–2.3 log units) for 30 and 60 L/min	Airborne *S. aureus*, *P. aeruginosa*, and *L. pneumophila* can be inactivated by the UV prototype device evaluated in this study, with *P. aeruginosa* as the most susceptible to UVGI.

									respectively) were significantly lower than those at low RH (4–4.3 and 3.7–4 log units) and medium RH (3.9– 4.2 and 2.9–3.3 log units) (both $p < 0.05$). K values for *S. aureus*, *P. aeruginosa*, and *L. pneumophila* over the RH of 12.7–90% were 0.7–7.0 × 10^4, 0.9–7.5 × 10^4, and 0.8–6.3 × 10^4 $cm^2/\mu W$ s respectively.	
19	Wood ME et al., 2018	Distance rig	*Pseudomonas aeruginosa*	From patients with the average concentration of 6.33 × 10^7 cfu/ml in their sputum samples.	Surgical and N95 face masks.	Coughs from patients.	6-stage Andersen impactor, pump was run at 28.3 L/min.	Agar plates were incubated at 37 °C for 72 h. *P. aeruginosa* identification was confirmed by oxidase testing, 42 °C growth, and matrix-assisted laser desorption/ionization–time-of-flight mass spectrometry. *P. aeruginosa* genotyping was undertaken using the iPLEX20SNP assay.	11% patients produced *P. aeruginosa*–positive aerosols while wearing the surgical mask, and 21% grew *P. aeruginosa* in their aerosol cultures when wearing the N95 mask. In contrast, 68% of these participants grew *P. aeruginosa* in their aerosols using cough etiquette and 76% produced aerosols containing viable *P. aeruginosa* in the uncovered cough maneuver.	Face masks reduce cough-generated P. aeruginosa aerosols, with the surgical mask providing enhanced comfort. Cough etiquette was less effective at reducing viable aerosols.
20	Davidson C et al., 2011	Chamber	*Bacillus anthracis*	10^6 - 10^8 cfu/mL	The 11 models included five surgical masks, three filtering face-piece disposable N95 respirators, and three surgical N95 respirators.	Six-jet Collison nebulizer, run for 8 min.	AGI-30 air samplers, airflow rate of 12.5 L/min. The AGI-30 sampler was fitted to the inside of the manikin head-form's mouth.	Tryptic soy agar (TSA) plate was used for spread plate enumeration.	Mean relative efficiency of respiratory protective equipment (RPE) was from 34 to 69%. Relative efficiency of surgical mask ranged 34-67%, of surgical N95 respirator: 34-62% and of N95: 66-69%.	Neither respiratory protective equipment type nor brand name was an indicator of respiratory protective equipment relative efficiency.

21	Davidson CS et al., 2013	Chamber	*Bacillus anthracis*	10^6 - 10^7 cfu/mL	11 models of N95 respirators and surgical masks. A manikin head form with N95 respirators or surgical masks, and manikin head form without N95 respirators or surgical masks were placed in the bioaerosol chamber.	Six-jet Collison nebulizer, run for 8 min.	An AGI-30 sampler filled with phosphate buffered water was fitted behind the mouth of each manikin head form to collect endospore bioaerosol samples. Samples were taken for 15 min.	Tryptic soy agar (TSA) plate was used for spread plate enumeration.	Geometric Mean (GM) relative efficiency of N95 respirators and surgical masks to aerosols *B. anthracis* ranged from 34–65%. N95 respirator had the highest GM relative efficiency.	This study further highlights the importance of fit and face seal leakage as factors in the respiratory protection provided by N95 respirators.
22	Zhao Y et al., 2014	Tunnels	*Enterococcus faecalis* and infectious bursal disease virus	7 and 9 $\log_{10}$ cfu/mL for *Enterococcus faecalis* and 6.7 log EID_{50}/mL for virus	The stainless-steel UV-PCO consisted of a rectangular contact cell with two funnels mounted on both sides. Three UV lamps, each had UV wattage of 11 W. Three UV irradiance levels could be created in the contact cell; that is, zero irradiance (all lamps turned off), low irradiance (middle lamp turned on), and high irradiance (two side lamps turned on).	Walther Pilot spray gun with a nozzle of 0.5 mm diameter.	MD-8 air samplers, air flow rate was 30 L/min for 2 min.	Liquid samples of *E. faecalis* was serially diluted, one tenth of a milliliter of each dilution was plated on a petri dish with sheep blood agar. The petri dishes were then incubated at 37 °C for 48 h.	The UV-PCO scrubber eliminated >99.7% of airborne *E. faecalis* under one UV lamp irradiance, and the reduction was further increased by 0.2–0.3% when the second UV lamp was added. The Z-value of airborne *E. faecalis* to UV irradiance was 9.3×10^4 cm^2 /μW s.	UV-PCO scrubber can be used as an effective and efficient technology for inactivating airborne bacteria and virus.
23	Pyrgiotakis G et al., 2014	Chamber	*Serratia marcescens*	$10^{4.5}$ cfu/mL	Engineered Water Nanostructures (EWNS) were synthesized by electro-spraying condensed atmospheric water vapor recovered from room air. A high voltage (5 kV) is then applied between the electrode and a grounded counter electrode placed 5 mm from the electrode. The strong electric field (10^7 V/m) between the two electrodes causes negative charges to accumulate on the surface of the condensed water.	A CN-6 Collison nebulizer, operated at 103 kPa (15 psi).	N6 single stage viable impactor, at 28.3 lpm, sampled for 1 min at 3-time intervals.	Tryptic soy agar (TSA) plates were incubated for 48 h.	The EWNS reduced the culturable airborne bacteria concentration, at steady state conditions, by 50% in comparison with the control experiments, and completely eradicated the culturable airborne bacteria during the decay portion of the experiment, in approximately 30 min, which is 50% less time compared to the control.	This novel, chemical free, nanotechnology-based method has the potential to be used to against airborne infectious diseases.

24	Lai ACK et al., 2016	Ductwork	*Escherichia coli*, *Pseudomonas alcaligenes*, *Staphylococcus epidermidis*, *Micrococcus luteus* and *Serratia marcescens*	10^8 -10^9 cfu in 50 mL sterilized distilled water.	A cold plasma unit of 4 W was used. The plasma discharge was based on a dielectric barrier mechanism. The unit had a plasma emission tube of 18 cm long and was installed according to manufacturer's recommendations. The unit can handle a maximal airflow rate up to 470 L/ s which was well above the maximum airflow rate of 280 L/s.	24-jet Collison nebulizer with inlet pressure of 275.8 kPa, operated at least 10 min before the experiment started.	Two single-stage viable Andersen cascade impactors at 2 locations, sampled for 106 s.	Nutrient agar or Trypticase soy agar, incubated at 30 °C for 24-72 h.	The inactivation efficacies for *E. coli*, *P. alcaligenes* and *S. epidermidis*, varied from 20% to 70%. No detectable inactivation effect was found for *M. luteus* and *S. marcescens*. The inactivation efficacy at 90% RH dropped to 10% of the value measured at 55% RH.	Cold plasma technology has high potential to be used as an energy-efficient method for air disinfection.
25	Yao M et al., 2005	Chamber	*Pseudomonas fluorescens*	N/A	Electrostatic fields of up to ±10 kV/cm. Bacteria exposure time of ~ 30 s.	Collison nebulizer, operated at a flow rate 2.5 L/min.	Bio Sampler, operated at 12.5 L/min for 5 min.	Trypticase soy agar plates were grown at 26 °C for 18 h.	No significant capturability difference was observed between the electrostatic field exposed and control bacteria.	The electrostatic fields were not effective in inactivating airborne bacteria.
26	Rudnick, S. N et al. 2009	Test room	*Serratia marcescens*	N/A	Portable in-duct UVGI apparatus contains 4 identical in-duct UV modules, each module has a 10 W cylindrical low-pressure mercury germicidal lamp. The airflow was adjusted at the rates of 50 and 100 cfm.	Six-jet Collison nebulizer.	Six-stage Andersen microbial impactors, operated at 28.3 L/min.	Nutrient agar was used, incubate at 37 °C for 2 days.	At an airflow rate of 100 cfm (0.047 m^3/s) with the lamps in only one or two modules powered, no bacteria were detected after passage through the apparatus. Additional tests with one module powered and 90% of the lamp covered, the inactivation rate of the portable in-duct UVGI apparatus at airflow rate 50 cfm (0.024 m^3/s) and 100 cfm (0.047 m^3/s) were 97.43 and 92.59% respectively.	Portable in-duct UVGI apparatus was effective in killing airborne bacteria.

Quality assessment

1. Wenke, C et al. 2017		
Intervention	Filter	
Microorganism	Equine arteritis virus; PRRSV; bovine enterovirus 1; *Actinobacillus pleuropneumoniae*; *Staphylococcus aureus*	
Device	Chamber	
Outcomes	Reduction rate	
**Quality Assessment**		
**Laboratory-based or chamber-based study**		**Author's judgement**
**Study design**	Lab based study/chamber study with other environmental conditions controlled to be consistent for all experiments	
	The intervention system/device has been used in other studies or has been peer-reviewed/in published paper	
**Method**	The intervention system/device	*
	Nebulizing method	*
	Collecting air samples	*
	Repeated experiments	*
	Microorganisms to be nebulized	*
	Lab methods	*
	Statistical method	*
**Result**	The effectiveness of the intervention method	*
	Comparison of data with p-value	*

2. Furuhashi, M et al. 1978		
Intervention	Commercial air filters	
Microorganism	*Staphylococcus aureus*, *Serratia marcescens*	
Device	Chamber	
Outcomes	Percentage of bacterial filtration efficiency %BFE	
**Quality Assessment**		
**Laboratory-based or chamber-based study**		**Author's judgement**
**Study design**	Lab based study/chamber study with other environmental conditions controlled to be consistent for all experiments	
	The intervention system/device has been used in other studies or has been peer-reviewed/in published paper	
**Method**	The intervention system/device	*
	Nebulizing method	*
	Collecting air samples	*
	Repeated experiments	
	Microorganisms to be nebulized	*
	Lab methods	*
	Statistical method	
**Result**	The effectiveness of the intervention method	*
	Comparison of data with p-value	*

3. Bergeron, V et al. 2011		
Intervention	High-throughput in-room air decontamination unit in conjunction with a standard AII room operated with and without negative pressure	
Microorganism	Non-pathogenic H5N2; H1N1 swine influenza virus; BCG *Mycobacterium bovis* and *S. marcescens*	
Device		
Outcomes	Reduction rate	
**Quality Assessment**		
**Laboratory-based or chamber-based study**		**Author's judgement**

**Study design**	Lab based study/chamber study with other environmental conditions controlled to be consistent for all experiments	*
	The intervention system/device has been used in other studies or has been peer-reviewed/in published paper	
**Method**	The intervention system/device	*
	Nebulizing method	*
	Collecting air samples	*
	Repeated experiments	
	Microorganisms to be nebulized	*
	Lab methods	
	Statistical method	
**Result**	The effectiveness of the intervention method	*
	Comparison of data with p-value	

**4. Sattar, S. A et al. 2016**		
Intervention	Commercial devices, combination of HEPA filter and UV light	
Microorganism	*Staphylococcus aureus*; *Klebsiella pneumoniae*	
Device	Chamber	
Outcomes	CFU	
**Quality Assessment**		
**Laboratory-based or chamber-based study**		**Author's judgement**
**Study design**	Lab based study/chamber study with other environmental conditions controlled to be consistent for all experiments	*
	The intervention system/device has been used in other studies or has been peer-reviewed/in published paper	
**Method**	The intervention system/device	*
	Nebulizing method	*
	Collecting air samples	*
	Repeated experiments	
	Microorganisms to be nebulized	*
	Lab methods	
	Statistical method	*
**Result**	The effectiveness of the intervention method	*
	Comparison of data with p-value	

**5. B. Zargar et al. 2018**		
Intervention	Commercial devices, combination of HEPA filter and UV light	
Microorganism	*Staphylococcus aureus* and *Acinobacter baumannii*; and a bacterial spore former (Geobacillus stearothermophilus)	
Device	Chamber	
Outcomes	Inactivation rate	
**Quality Assessment**		
**Laboratory-based or chamber-based study**		**Author's judgement**
**Study design**	Lab based study/chamber study with other environmental conditions controlled to be consistent for all experiments	*
	The intervention system/device has been used in other studies or has been peer-reviewed/in published paper	
**Method**	The intervention system/device	*
	Nebulizing method	*
	Collecting air samples	*
	Repeated experiments	*
	Microorganisms to be nebulized	*
	Lab methods	*
	Statistical method	
**Result**	The effectiveness of the intervention method	*
	Comparison of data with p-value	

6. Lisa Eisenloffel et al. 2019		
Intervention	Commercial devices, combination of air filter and UV light	
Microorganism	*Staphylococcus aureus*, *Actinobacillus pleuropneumoniae*, porcine parvovirus (PPV) and porcine reproductive and respiratory syndrome virus	
Device	Chamber	
Outcomes	Inactivation rate	
**Quality Assessment**		
**Laboratory-based or chamber-based study**		**Author's judgement**
**Study design**	Lab based study/chamber study with other environmental conditions controlled to be consistent for all experiments	*
	The intervention system/device has been used in other studies or has been peer-reviewed/in published paper	
**Method**	The intervention system/device	*
	Nebulizing method	*
	Collecting air samples	*
	Repeated experiments	
	Microorganisms to be nebulized	*
	Lab methods	
	Statistical method	*
**Result**	The effectiveness of the intervention method	*
	Comparison of data with p-value	

7. Li, C. S et al. 2003		
Intervention	UV-C and titanium dioxide photocatalyst	
Microorganism	*Legionella pneumoniae*	
Device	Chamber	
Outcomes	Rate of reduction	
**Quality Assessment**		
**Laboratory-based or chamber-based study**		**Author's judgement**
**Study design**	Lab based study/chamber study with other environmental conditions controlled to be consistent for all experiments	
	The intervention system/device has been used in other studies or has been peer-reviewed/in published paper	
**Method**	The intervention system/device	*
	Nebulizing method	*
	Collecting air samples	*
	Repeated experiments	
	Microorganisms to be nebulized	*
	Lab methods	*
	Statistical method	
**Result**	The effectiveness of the intervention method	*
	Comparison of data with p-value	

8. King, B et al. 2011		
Intervention	UV-C	
Microorganism	*Bacillus atrophaeus*; *Pantoea agglomerans* and *Yersinia ruckeri*	
Device	Chamber	
Outcomes	Rate of reduction	
**Quality Assessment**		
**Laboratory-based or chamber-based study**		**Author's judgement**
**Study design**	Lab based study/chamber study with other environmental conditions controlled to be consistent for all experiments	

	The intervention system/device has been used in other studies or has been peer-reviewed/in published paper	
**Method**	The intervention system/device	*
	Nebulizing method	*
	Collecting air samples	*
	Repeated experiments	*
	Microorganisms to be nebulized	*
	Lab methods	*
	Statistical method	
**Result**	The effectiveness of the intervention method	*
	Comparison of data with p-value	

**9. Riley, R. L et al. 1976**		
Intervention	UV-C	
Microorganism	*Mycobacterium bovis* BCG; *M. tuberculosis* and *M. phlei*	
Device	Test room	
Outcomes	Z value	
**Quality Assessment**		
**Laboratory-based or chamber-based study**		**Author's judgement**
**Study design**	Lab based study/chamber study with other environmental conditions controlled to be consistent for all experiments	
	The intervention system/device has been used in other studies or has been peer-reviewed/in published paper	
**Method**	The intervention system/device	*
	Nebulizing method	*
	Collecting air samples	*
	Repeated experiments	
	Microorganisms to be nebulized	*
	Lab methods	*
	Statistical method	
**Result**	The effectiveness of the intervention method	*
	Comparison of data with p-value	

**10. Ko, G et al. 2000**		
Intervention	UV-C	
Microorganism	*Serratia marcescens* and *Mycobacterium bovis* BCG	
Device	Chamber	
Outcomes	CFU, Z value	
**Quality Assessment**		
**Laboratory-based or chamber-based study**		**Author's judgement**
**Study design**	Lab based study/chamber study with other environmental conditions controlled to be consistent for all experiments	
	The intervention system/device has been used in other studies or has been peer-reviewed/in published paper	
**Method**	The intervention system/device	*
	Nebulizing method	*
	Collecting air samples	*
	Repeated experiments	
	Microorganisms to be nebulized	*
	Lab methods	*
	Statistical method	*
**Result**	The effectiveness of the intervention method	*
	Comparison of data with p-value	

11. Peccia, J et al. 2001		
Intervention	UV-C	
Microorganism	*Serratia marcescens*, *Bacillus subtilis*, and *Mycobacterium parafortuitum*	
Device	Chamber	
Outcomes	Rate of reduction	
**Quality Assessment**		
**Laboratory-based or chamber-based study**		**Author's judgement**
**Study design**	Lab based study/chamber study with other environmental conditions controlled to be consistent for all experiments	*
	The intervention system/device has been used in other studies or has been peer-reviewed/in published paper	
**Method**	The intervention system/device	*
	Nebulizing method	*
	Collecting air samples	*
	Repeated experiments	*
	Microorganisms to be nebulized	*
	Lab methods	
	Statistical method	
**Result**	The effectiveness of the intervention method	*
	Comparison of data with p-value	

12. Peccia, J et al. 2004		
Intervention	UV-C	
Microorganism	*Mycobacterium bovis* bacillus Calmette-Guerin (BCG)	
Device	Lab	
Outcomes	Rate of reduction or CFU	
**Quality Assessment**		
**Laboratory-based or chamber-based study**		**Author's judgement**
**Study design**	Lab based study/chamber study with other environmental conditions controlled to be consistent for all experiments	*
	The intervention system/device has been used in other studies or has been peer-reviewed/in published paper	
**Method**	The intervention system/device	*
	Nebulizing method	*
	Collecting air samples	*
	Repeated experiments	
	Microorganisms to be nebulized	*
	Lab methods	*
	Statistical method	*
**Result**	The effectiveness of the intervention method	*
	Comparison of data with p-value	

13. Yang, Y et al. 2018		
Intervention	UV-C	
Microorganism	*Serratia marcescens*, *Pseudomonas alcaligenes*, *Escherichia coli*, *Salmonella enterica*, and *Staphylococcus epidermidis*	
Device	Chamber	
Outcomes	Inactivation rate, Z value	
**Quality Assessment**		
**Laboratory-based or chamber-based study**		**Author's judgement**
**Study design**	Lab based study/chamber study with other environmental conditions controlled to be consistent for all experiments	*
	The intervention system/device has been used in other studies or has been peer-reviewed/in published paper	
**Method**	The intervention system/device	*

	Nebulizing method	*
	Collecting air samples	*
	Repeated experiments	
	Microorganisms to be nebulized	*
	Lab methods	
	Statistical method	
**Result**	The effectiveness of the intervention method	*
	Comparison of data with p-value	

**14. Zhang, H et al. 2020**		
Intervention	UV light	
Microorganism	*Staphylococcus epidermidis*, *Pseudomonas alcaligenes*, and *Escherichia coli*	
Device	Duct system	
Outcomes	Z values	
**Quality Assessment**		
**Laboratory-based or chamber-based study**		**Author's judgement**
**Study design**	Lab based study/chamber study with other environmental conditions controlled to be consistent for all experiments	
	The intervention system/device has been used in other studies or has been peer-reviewed/in published paper	
**Method**	The intervention system/device	*
	Nebulizing method	*
	Collecting air samples	*
	Repeated experiments	
	Microorganisms to be nebulized	*
	Lab methods	*
	Statistical method	*
**Result**	The effectiveness of the intervention method	*
	Comparison of data with p-value	

**15. Nunayon, S.S et al. 2019**		
Intervention	UV light LED	
Microorganism	*Escherichia coli*, *Serratia marcescens*, and *Staphylococcus epidermis*	
Device	Test room	
Outcomes	Inactivation rate	
**Quality Assessment**		
**Laboratory-based or chamber-based study**		**Author's judgement**
**Study design**	Lab based study/chamber study with other environmental conditions controlled to be consistent for all experiments	
	The intervention system/device has been used in other studies or has been peer-reviewed/in published paper	
**Method**	The intervention system/device	*
	Nebulizing method	*
	Collecting air samples	*
	Repeated experiments	
	Microorganisms to be nebulized	*
	Lab methods	*
	Statistical method	
**Result**	The effectiveness of the intervention method	*
	Comparison of data with p-value	

**16. Xu, P et al. 2003**	
Intervention	UV-C
Microorganism	*Bacillus subtilis* (spores), *Mycobacterium parafortuitum*, and *Mycobacterium bovis* BCG

Device	Test room	
Outcomes	Rate of reduction; Z value	
**Quality Assessment**		
**Laboratory-based or chamber-based study**		**Author's judgement**
**Study design**	Lab based study/chamber study with other environmental conditions controlled to be consistent for all experiments	*
	The intervention system/device has been used in other studies or has been peer-reviewed/in published paper	
**Method**	The intervention system/device	*
	Nebulizing method	*
	Collecting air samples	*
	Repeated experiments	
	Microorganisms to be nebulized	*
	Lab methods	*
	Statistical method	
**Result**	The effectiveness of the intervention method	*
	Comparison of data with p-value	

**17. Ko, G et al. 2002**		
Intervention	UV-C	
Microorganism	*Serratia marcescens* and *Mycobacterium bovis*	
Device	Chamber	
Outcomes	Rate of reduction	
**Quality Assessment**		
**Laboratory-based or chamber-based study**		**Author's judgement**
**Study design**	Lab based study/chamber study with other environmental conditions controlled to be consistent for all experiments	*
	The intervention system/device has been used in other studies or has been peer-reviewed/in published paper	
**Method**	The intervention system/device	*
	Nebulizing method	*
	Collecting air samples	*
	Repeated experiments	
	Microorganisms to be nebulized	*
	Lab methods	
	Statistical method	*
**Result**	The effectiveness of the intervention method	*
	Comparison of data with p-value	

**18. Chang, C. W et al. 2013**		
Intervention	UV-C	
Microorganism	*Staphylococcus aureus, Pseudomonas aeruginosa* and *Legionella pneumophila*	
Device	Chamber	
Outcomes	Log of reduction	
**Quality Assessment**		
**Laboratory-based or chamber-based study**		**Author's judgement**
**Study design**	Lab based study/chamber study with other environmental conditions controlled to be consistent for all experiments	*
	The intervention system/device has been used in other studies or has been peer-reviewed/in published paper	
**Method**	The intervention system/device	*
	Nebulizing method	*
	Collecting air samples	*
	Repeated experiments	
	Microorganisms to be nebulized	*
	Lab methods	*

	Statistical method	*
**Result**	The effectiveness of the intervention method	*
	Comparison of data with p-value	

**19. Michelle, E. W et al. 2018**		
Intervention	Other method: mask	
Microorganism	*Pseudomonas aeruginosa*	
Device	Chamber	
Outcomes	Reduction in aerosol Pa load	
**Quality Assessment**		
**Laboratory-based or chamber-based study**		**Author's judgement**
**Study design**	Lab based study/chamber study with other environmental conditions controlled to be consistent for all experiments	*
	The intervention system/device has been used in other studies or has been peer-reviewed/in published paper	*
**Method**	The intervention system/device	*
	Nebulizing method/air produced by patients	*
	Collecting air samples	*
	Repeated experiments (25 patients)	*
	Microorganisms to be nebulized	
	Lab methods	*
	Statistical method	*
**Result**	The effectiveness of the intervention method	*
	Comparison of data with p-value	*

**20. Davidson, C et al. 2011**		
Intervention	Other method: N95 respirator and surgical mask	
Microorganism	*Bacillus anthracis*	
Device	Lab	
Outcomes	Relative efficiency	
**Quality Assessment**		
**Laboratory-based or chamber-based study**		**Author's judgement**
**Study design**	Lab based study/chamber study with other environmental conditions controlled to be consistent for all experiments	
	The intervention system/device has been used in other studies or has been peer-reviewed/in published paper	
**Method**	The intervention system/device	*
	Nebulizing method	*
	Collecting air samples	*
	Repeated experiments	
	Microorganisms to be nebulized	*
	Lab methods	*
	Statistical method	*
**Result**	The effectiveness of the intervention method	*
	Comparison of data with p-value	

**21. Davidson, C et al. 2013**	
Intervention	Other method: N95 respirator and surgical mask
Microorganism	*Bacillus anthracis* endospore
Device	Chamber
Outcomes	Relative efficiency
**Quality Assessment**	

Laboratory-based or chamber-based study		Author's judgement
Study design	Lab based study/chamber study with other environmental conditions controlled to be consistent for all experiments	
	The intervention system/device has been used in other studies or has been peer-reviewed/in published paper	
Method	The intervention system/device	*
	Nebulizing method	*
	Collecting air samples	*
	Repeated experiments	
	Microorganisms to be nebulized	*
	Lab methods	*
	Statistical method	*
Result	The effectiveness of the intervention method	*
	Comparison of data with p-value	

22. Zhao, Y et al. 2014		
Intervention	Ultraviolet photocatalytic oxidation scrubber	
Microorganism	*Enterococcus faecalis* and infectious bursal disease viruses	
Device	Lab	
Outcomes	Rate of elimination	
**Quality Assessment**		
**Laboratory-based or chamber-based study**		**Author's judgement**
**Study design**	Lab based study/chamber study with other environmental conditions controlled to be consistent for all experiments	*
	The intervention system/device has been used in other studies or has been peer-reviewed/in published paper	
**Method**	The intervention system/device	*
	Nebulizing method	*
	Collecting air samples	*
	Repeated experiments	
	Microorganisms to be nebulized	*
	Lab methods	*
	Statistical method	*
**Result**	The effectiveness of the intervention method	*
	Comparison of data with p-value	*

23.Pygiotakis, G et al. 2014		
Intervention	Other method: engineered water nanostructures	
Microorganism	*Serratia marcescens*	
Device	Chamber	
Outcomes	Rate of reduction or CFU	
**Quality Assessment**		
**Laboratory-based or chamber-based study**		**Author's judgement**
**Study design**	Lab based study/chamber study with other environmental conditions controlled to be consistent for all experiments	*
	The intervention system/device has been used in other studies or has been peer-reviewed/in published paper	
**Method**	The intervention system/device	*
	Nebulizing method	*
	Collecting air samples	*
	Repeated experiments	
	Microorganisms to be nebulized	*
	Lab methods	
	Statistical method	*
**Result**	The effectiveness of the intervention method	*

	Comparison of data with p-value	

**24. Lai, A. C. K et al. 2016**		
Intervention	Other method: cold plasma	
Microorganism	*Escherichia coli*; *Pseudomonas alcaligenes*; *Staphylococcus epidermidis*; *Micrococcus luteus* and *Serratia marcescens*	
Device	Ductwork system	
Outcomes	Inactivation efficacy	
**Quality Assessment**		
**Laboratory-based or chamber-based study**		**Author's judgement**
**Study design**	Lab based study/chamber study with other environmental conditions controlled to be consistent for all experiments	*
	The intervention system/device has been used in other studies or has been peer-reviewed/in published paper	
**Method**	The intervention system/device	*
	Nebulizing method	*
	Collecting air samples	*
	Repeated experiments	
	Microorganisms to be nebulized	*
	Lab methods	
	Statistical method	*
**Result**	The effectiveness of the intervention method	*
	Comparison of data with p-value	

**25. Yao, M et al. 2005**		
Intervention	Other method: electrostatic field	
Microorganism	*Pseudomonas flourescens* and *Bacillus subtilis* var. niger	
Device	Chamber	
Outcomes	Inactivated rate	
**Quality Assessment**		
**Laboratory-based or chamber-based study**		**Author's judgement**
**Study design**	Lab based study/chamber study with other environmental conditions controlled to be consistent for all experiments	*
	The intervention system/device has been used in other studies or has been peer-reviewed/in published paper	
**Method**	The intervention system/device	
	Nebulizing method	*
	Collecting air samples	*
	Repeated experiments	*
	Microorganisms to be nebulized	*
	Lab methods	*
	Statistical method	
**Result**	The effectiveness of the intervention method	*
	Comparison of data with p-value	*

**26. Rudnick, S. N et al. 2009**		
Intervention	Portable in duct UVGI	
Microorganism	*Bacillus atrophaeus* Nukamura and *Serratia marcescens;*	
Device	Lab and buildings	
Outcomes	Inactivating efficiency	
**Quality Assessment**		
**Laboratory-based or chamber-based study**		**Author's judgement**
**Study design**	Lab based study/chamber study with other environmental conditions controlled to be consistent for all experiments	

	The intervention system/device has been used in other studies or has been peer-reviewed/in published paper	
**Method**	The intervention system/device	*
	Nebulizing method	*
	Collecting air samples	*
	Repeated experiments	
	Microorganisms to be nebulized	*
	Lab methods	*
	Statistical method	
**Result**	The effectiveness of the intervention method	*
	Comparison of data with p-value	

Studies excluded due to not passing the quality assessment

**1. Kundsin, R. B et al. 1966**	
Intervention	UV-C
Microorganism	Mycoplasma: M. hominis II, *Mycoplasma pharyngis*, *Mycoplasma pneumoniae*
Device	Chamber
Outcomes	Inactivation rate
**Quality Assessment**	

**Laboratory-based or chamber-based study**		**Author's judgement**
**Study design**	Lab based study/chamber study with other environmental conditions controlled to be consistent for all experiments	
	The intervention system/device has been used in other studies or has been peer-reviewed/in published paper	
**Method**	The intervention system/device	*
	Nebulizing method	*
	Collecting air samples	
	Repeated experiments	
	Microorganisms to be nebulized	*
	Lab methods	
	Statistical method	
**Result**	The effectiveness of the intervention method	*
	Comparison of data with p-value	

**2. Lai, M. H et al. 2003**	
Intervention	UV-C: portable UV-C device
Microorganism	*Staphylococcus aureus*
Device	Chamber
Outcomes	CFU
**Quality Assessment**	

**Laboratory-based or chamber-based study**		**Author's judgement**
**Study design**	Lab based study/chamber study with other environmental conditions controlled to be consistent for all experiments	
	The intervention system/device has been used in other studies or has been peer-reviewed/in published paper	
**Method**	The intervention system/device	*
	Nebulizing method	
	Collecting air samples	*
	Repeated experiments	
	Microorganisms to be nebulized	*
	Lab methods	*
	Statistical method	
**Result**	The effectiveness of the intervention method	*
	Comparison of data with p-value	

3. Welch, D et al. 2018		
Intervention	UV-C	
Microorganism	Methicillin-resistant *Staphylococcus aureus* (MRSA) and influenza A virus	
Device	Chamber	
Outcomes	Inactivation rate	
**Quality Assessment**		
**Laboratory-based or chamber-based study**		**Author's judgement**
**Study design**	Lab based study/chamber study with other environmental conditions controlled to be consistent for all experiments	*
	The intervention system/device has been used in other studies or has been peer-reviewed/in published paper	
**Method**	The intervention system/device	
	Nebulizing method	
	Collecting air samples	*
	Repeated experiments	
	Microorganisms to be nebulized	*
	Lab methods	
	Statistical method	
**Result**	The effectiveness of the intervention method	*
	Comparison of data with p-value	
